# Myositis-Associated Interstitial Lung Disease Presenting as Acute Respiratory Distress Syndrome: A Retrospective Observational Study

**DOI:** 10.3390/jcm15062336

**Published:** 2026-03-18

**Authors:** Sung Won Chang, Sang Hyuk Kim, Juwhan Choi, Jee Youn Oh, Kyung Hoon Min, Gyu Young Hur, Hwan Seok Yong, Sung Yong Lee, Jae Jeong Shim, Jae Kyeom Sim

**Affiliations:** 1 Division of Pulmonary, Allergy, and Critical Care Medicine, Department of Internal Medicine, Korea University Guro Hospital, Korea University College of Medicine, Seoul 08308, Republic of Korea; lego42st@gmail.com (S.W.C.);; 2 Department of Radiology, Korea University Guro Hospital, Korea University College of Medicine, Seoul 08308, Republic of Korea

**Keywords:** interstitial lung disease, myositis, acute respiratory distress syndrome

## Abstract

**Background/Objectives**: Myositis-associated interstitial lung disease (ILD) can occasionally present as acute respiratory distress syndrome (ARDS); however, clinical data on this presentation remain limited. This study aimed to describe the clinical characteristics and outcomes of patients with myositis-associated ILD presenting as ARDS. **Methods**: We conducted a single-center retrospective observational study of patients with myositis-associated ILD who were admitted to the intensive care unit (ICU) for acute hypoxemic respiratory failure. **Results**: Ten patients positive for myositis-specific antibodies met the new global ARDS definition. The median age was 62 years, and eight patients were male. Antibody profiles included anti-MDA-5 (*n* = 5), anti-synthetase antibodies (Jo-1 [*n* = 1], PL-7 [*n* = 2], EJ [*n* = 4]), and NXP-2 (*n* = 1). Fever and cutaneous manifestations were the most common extrapulmonary features. Chest computed tomography demonstrated diffuse alveolar damage patterns in six patients and organizing pneumonia patterns in four. At ICU admission, four patients required mechanical ventilation and six received high-flow nasal cannula, of whom four subsequently progressed to mechanical ventilation. Extracorporeal membrane oxygenation was implemented in three patients. All patients received high-dose corticosteroids, six underwent steroid pulse therapy, and four additionally received immunosuppressive agents. Six patients died during hospitalization. **Conclusions**: Myositis-associated ILD may present as ARDS and should be considered in patients with ARDS of unclear etiology. Careful physical examination and autoantibody testing may assist in recognizing this condition in the critical care setting.

## 1. Introduction

Acute respiratory distress syndrome (ARDS) is defined as acute hypoxemic respiratory failure that develops or worsens within one week, and is accompanied by bilateral opacities on chest imaging [1]. As implied by the term, ARDS represents a clinical syndrome rather than a specific disease entity. Accordingly, improving clinical outcomes requires not only general supportive care but also identification of the underlying cause and initiation of appropriate targeted therapy.

Although uncommon, connective tissue disease-associated interstitial lung disease (CTD-ILD) should be considered as a potential etiology of ARDS [2,3]. In a large ARDS cohort, 9.5% of patients had no identifiable risk factor at the time of diagnosis; however, only 5.1% of this subgroup underwent immunologic testing [4]. These findings suggest that CTD-ILD presenting as ARDS may be substantially underrecognized in clinical practice.

Idiopathic inflammatory myopathy (IIM), a heterogeneous group of autoimmune diseases, represents a notable example. Although the formal diagnostic criteria primarily include muscle inflammation, weakness, and characteristic skin manifestations [5], a broad spectrum of extramuscular manifestation may also occur. In patients with IIM, pulmonary involvement is common, and ILD may precede or coincide with the onset of muscle or skin manifestations [6,7,8]. Furthermore, some patients with ILD do not meet the formal diagnostic criteria for IIM [5], yet exhibit clinical manifestations similar to IIM, including muscle and skin involvement, and test positive for relevant autoantibodies, such as myositis-specific antibodies (MSAs) or myositis-associated antibodies [9]. Accurate diagnosis and management of myositis-associated ILD in these patients are particularly challenging [10,11]. The rapidly progressive form of myositis-associated ILD poses significant diagnostic difficulties, especially in distinguishing it from other causes of ARDS. In the absence of a known history of ILD or CTD, clinicians may fail to recognize ILD as a potential underlying cause of ARDS. In addition, the low prevalence and limited clinical awareness of myositis-associated ILD further hinder timely diagnosis and effective treatment [12].

This study aimed to characterize the clinical presentation and treatment responses of patients with myositis-associated ILD presenting with ARDS.

## 2. Materials and Methods

### 2.1. Study Design and Population

This retrospective observational study was conducted at a university-affiliated hospital in South Korea. Among patients with positive MSAs identified between January 2022 and December 2024, those who developed acute hypoxemic respiratory failure requiring ICU admission were retrospectively analyzed. Inclusion criteria were as follows: (1) age 19 years or older, (2) admission to the intensive care unit (ICU) for acute hypoxemic respiratory failure requiring respiratory support such as high-flow nasal cannula (HFNC) or mechanical ventilation, and (3) new or worsening pulmonary infiltrates on chest imaging. Exclusion criteria included: (1) patients with predominantly extrapulmonary manifestations, (2) patients who did not require ICU care, and (3) patients with a preexisting diagnosis of CTD or ILD before the onset of acute respiratory failure.

The MSAs evaluated in this study were as follows: anti-aminoacyl-tRNA synthetase (ARS) antibodies (Jo-1, PL-7, PL-12, EJ, and OJ) and antibodies to SRP, Mi-2α, melanoma differentiation-associated gene 5 (MDA-5), TIF1γ, and NXP-2. MSA testing was performed at the discretion of the attending physicians. In clinical practice, testing was generally considered when myositis was clinically suspected or when imaging findings were suggestive of ILD. The antibodies were measured using either a single-analyte anti-Jo-1 assay or a commercial Myositis-Specific 11 Antibody Panel (QuestDiagnostics, Secaucus, NJ, USA).

### 2.2. Data Collection

Data regarding demographics, clinical manifestations, laboratory tests, microbiological investigations, and radiologic features at or near the time of ICU admission were systematically extracted from electronic medical records. All chest computed tomography (CT) images were independently re-evaluated by a radiologist with over 20 years of experience in thoracic imaging to classify ILD patterns based on established radiologic descriptions of interstitial pneumonias [13]. Management details, including pharmacologic therapies, respiratory support, and clinical outcomes, were systematically recorded.

The eligibility of patients for anti-synthetase syndrome (ASS) and IIM was assessed according to established classification criteria (Connors’ and Solomon’s criteria for ASS [14,15], and the 2017 EULAR/ACR classification criteria for IIM [5]). The diagnosis and severity of ARDS were evaluated in accordance with the recently proposed new global ARDS definition, which includes patients receiving HFNC oxygen therapy at flow rates ≥ 30 L/min. According to this definition, acute onset or worsening of hypoxemic respiratory failure occurs within 1 week of the onset of a predisposing risk factor or new or worsening respiratory symptoms [1]. The possibility of cardiogenic pulmonary edema as the primary cause of pulmonary edema was assessed based on the overall clinical assessment, chest CT findings, and transthoracic echocardiography when available.

### 2.3. Statistical Analysis

This study was descriptive in nature. Continuous variables were summarized as medians and interquartile ranges (IQRs), and categorical variables were expressed as frequencies and percentages. Given the small sample size and the absence of a comparison group, formal inferential statistical testing was not applicable. All descriptive statistical calculations were performed using Microsoft Excel (version 2019, Microsoft Corporation, Redmond, WA, USA).

## 3. Results

**Figure 1:**
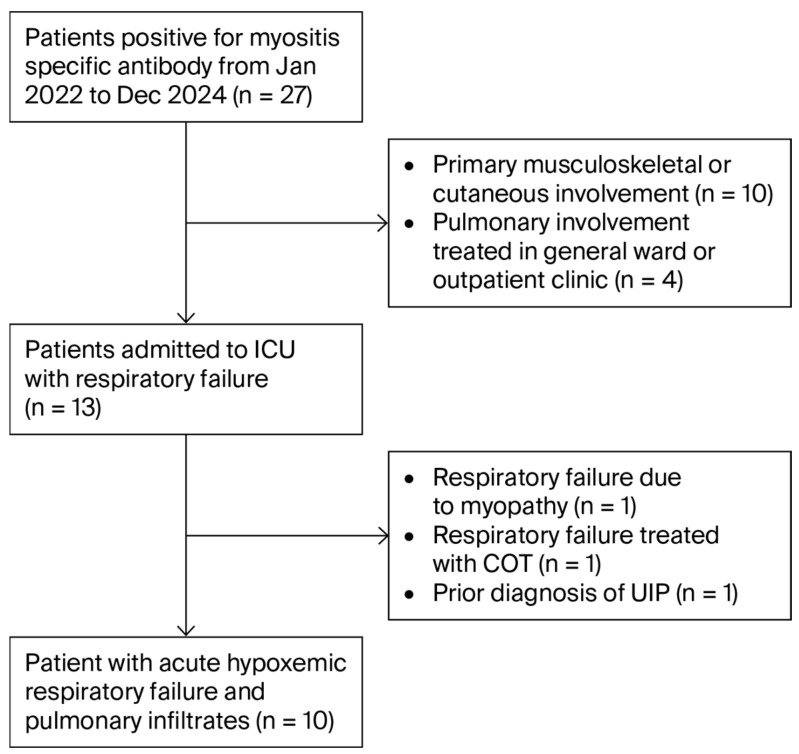
Flow chart. ICU, intensive care unit; COT, conventional oxygen therapy; UIP, usual interstitial pneumonia.

During the study period, 27 patients tested positive for MSAs. Fourteen were excluded as their primary clinical manifestations were extrapulmonary or they did not require ICU care. Among the remaining patients, three were further excluded: one required invasive mechanical ventilation due to respiratory failure solely from muscle weakness, one received only conventional oxygen therapy, and one had a previous diagnosis of ILD (probable UIP pattern) presenting with an acute exacerbation. Ultimately, 10 patients were included in the analysis (Figure 1).

### 3.1. Demographic and Clinical Features

The median age of the study population was 61.5 years (IQRs, 59.3~70.8), and 8 patients (80%) were male. Five patients (50%) had pre-existing comorbidities, including cardiac disease, diabetes, or liver cirrhosis. Two patients (20%) were transferred from other hospitals; neither had a prior diagnosis of lung disease or connective tissue disease, but both were clinically suspected of having CTD-ILD at the time of referral.

Anti-MDA-5 antibody was the most prevalent MSA, detected in 5 patients (50%). ARS antibodies were identified in 4 patients (40%), comprising anti-PL-7 (*n* = 2), anti-Jo-1 (*n* = 1), and anti-EJ (*n* = 1). Anti-NXP-2 antibody was present in one patient, and anti-Mi-2α antibody was detected in another patient, who also tested positive for anti-MDA-5.

With the exception of one patient who was unable to communicate due to aphasia, all patients reported dyspnea. Fever (≥37.8 °C) occurred in 6 patients (60%). Cutaneous manifestations were observed in 4 patients (40%), predominantly involving acral areas. These manifestations included mildly puffy hands with erythematous changes over the fingertips and joints consistent with Gottron’s papules; pruritic violaceous-to-erythematous eczematoid eruptions on the scalp, trunk, and extremities; digital and palmar rashes with ulcerative finger lesions and mechanic’s hands; and periungual erythema. Myalgia and muscle weakness were reported in 3 patients (30%) and 2 patients (20%), respectively (Table 1). All ARS-positive patients met the Connors criteria for ASS, and one patient with anti-Jo-1 antibody additionally fulfilled the Solomon criteria and the 2017 EULAR/ACR criteria for idiopathic inflammatory myopathy.

### 3.2. Laboratory Findings

C-reactive protein (CRP) levels were elevated in all patients, with a median value of 11.4 mg/L (IQRs, 7.8–17.8). The median procalcitonin level was 0.165 ng/mL (IQR, 0.08–0.405). Krebs von den Lungen-6 (KL-6) was measured in 7 patients and was elevated in 6, with a median level of 1048 U/mL (IQRs, 591–1380). Creatine phosphokinase was elevated in 2 patients but did not correlate with the presence of myalgia or muscle weakness. Anti-nuclear antibodies (ANA) assessed by indirect immunofluorescence were positive in 3 patients (30%), with low titers (1:40–1:160) showing speckled or homogeneous nuclear patterns. Autoantibodies other than MSAs were assessed in 7 patients, and 3 (43%) were positive for anti-SSA antibody. Bronchoalveolar lavage (BAL) was performed in 7 patients; all showed an increased proportion of neutrophils, and lymphocytosis was observed in 2 patients. Laboratory findings are summarized in Table 2.

### 3.3. Microbiological Evaluation

Comprehensive microbiological evaluation for pulmonary infections was performed as part of routine clinical care, and the results are summarized in Table 3. Blood cultures were obtained in all patients and showed no growth. Bronchoscopy was performed in nine patients, while sputum specimens were obtained in one patient without bronchoscopy. *Klebsiella pneumoniae* was isolated from a BAL specimen in one patient. Respiratory bacterial polymerase chain reaction (PCR) assays detected *Haemophilus influenzae* in one patient and both *Streptococcus pneumoniae* and *H. influenzae* in another patient. Respiratory viral PCR assays were performed in nine patients and all results were negative. Urinary antigen testing for *S. pneumoniae* was performed in all patients and for *Legionella pneumophila* in nine patients, and all results were negative.

*Pneumocystis jirovecii* PCR was positive in 3 patients (30%). Of these, one had received prolonged low-dose corticosteroid therapy, one had received high-dose corticosteroids for one week prior to BAL sampling, and one had not been exposed to immunosuppressive agents. β-D-glucan testing was performed in two of these patients, yielding values of 38 pg/mL and <10 pg/mL, both below the institutional positivity threshold of 60 pg/mL.

All patients received empirical antibiotics, and those who were positive for *Pneumocystis jirovecii* received appropriate additional treatment.

### 3.4. Radiologic Findings

Ground-glass opacities were evident in all patients. Consolidations appeared in 8 patients (80%), while fibrotic changes were noted in 5 (50%). Pulmonary lesions involved both lungs and were predominantly distributed in the bilateral lower lobes or peripheral regions. Based on radiologic ILD patterns, the diffuse alveolar damage (DAD) pattern was most frequent (60%), followed by an organizing pneumonia (OP) pattern (10%) and overlapping OP/non-specific interstitial pneumonia pattern (NSIP) (30%). Additional radiological information is provided in Table 4.

### 3.5. Management and Outcomes

The median ratio of the partial pressure of arterial oxygen to the fraction of inspired oxygen (P/F ratio) at ICU admission in the overall patients was 125.5 mmHg (IQRs, 105.5–146.8), and the median Sequential Organ Failure Assessment score at ICU admission was 3.5 (IQRs, 2.0–6.8). Four patients (40%) were mechanically ventilated at ICU admission, with an initial positive end-expiratory pressure ranging from 5 to 6 cmH_2_O. Their median ratio of the partial pressure of arterial oxygen to the fraction of inspired oxygen (P/F ratio) was a 122 mmHg (IQRs, 99.5–130.3), corresponding to moderate ARDS in three and severe ARDS in one. Two of these four patients (50%) required extracorporeal membrane oxygenation (ECMO): one ultimately underwent lung transplantation at another institution, whereas the other died while receiving ECMO. Of the two mechanically ventilated patients who did not receive ECMO, one died.

Six patients (60%) initially received HFNC support in the ICU, with flow rates ranging from 40 to 60 L/min. Their median P/F ratio was 136.5 mmHg (IQRs, 107.3–167.3), and all fulfilled the new global ARDS definition. Four of these six patients (67%) eventually required mechanical ventilation, including one who subsequently received ECMO. All patients who progressed from HFNC to mechanical ventilation died.

**Figure 2:**
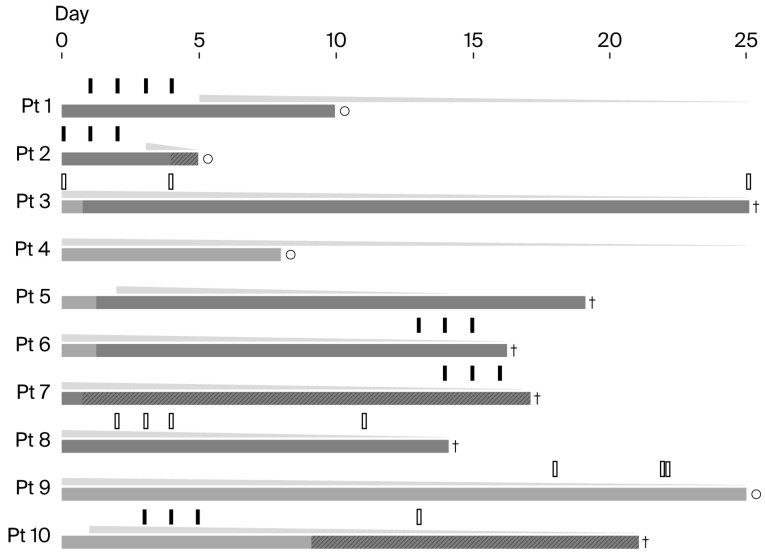
Patient-level clinical timelines in intensive care unit (ICU). Time is shown as days since ICU admission (day 0). Each horizontal row represents an individual patient. Horizontal bars indicate respiratory support, with light gray bars representing high-flow nasal cannula and dark gray bars representing invasive mechanical ventilation. Hatched dark gray bars indicate invasive mechanical ventilation with concomitant extracorporeal membrane oxygenation. Triangles above the bars indicate high-dose corticosteroid therapy with subsequent tapering. Vertical markers above the triangles indicate initiation of immunosuppressive therapy. Solid bars denote steroid pulse therapy (the number of solid bars indicates the number of pulses), whereas open bars denote other immunosuppressive agents. Crosses and open circles adjacent to the horizontal bars indicate death and survival at the end of the ICU course, respectively. The timeline is truncated at day 25; bars extending to the right margin indicate ongoing respiratory support or immunosuppressive therapy beyond the displayed time window.

High-dose corticosteroids were administered to all patients. Steroid pulse therapy (250–1000 mg/day) was used in 6 patients (60%), either prior to high-dose corticosteroids (*n* = 2), with pulse therapy initiated 0 and 1 day after ICU admission (median, 0.5 day), or as supplementary therapy during high-dose treatment (*n* = 4), with a median interval of 8 days (IQRs, 2.8–13.3) from ICU admission to pulse therapy initiation; all patients in the latter group died. Additional immunosuppressive agents were initiated in 4 patients (40%), with initiation occurring within 1 week (*n* = 1), within 2 weeks (*n* = 2), or after 2 weeks (*n* = 1). In the last patient, treatment was discontinued shortly after initiation due to the detection of *Pseudomonas aeruginosa*. Detailed data on management and outcomes are summarized in Table 5, and the corresponding patient-level clinical timelines illustrating respiratory support changes and immunomodulatory treatment initiation are presented in Figure 2.

## 4. Discussion

We described the clinical features of patients with myositis-associated ILD who developed acute hypoxemic respiratory failure requiring respiratory support. According to the new global ARDS definition, all patients met the diagnostic criteria [1]. Prior studies largely reported a chronic disease course in these patients [16,17], and research addressing presentation with acute respiratory failure has been sparse [18]. Currently, there remains no universally accepted consensus on the diagnosis of myositis-associated ILD [10]. When several myositis-related diagnostic criteria were applied [5,14,15], the patients fulfilled these classifications inconsistently, reflecting the diagnostic challenges encountered in clinical practice. Such diagnostic uncertainty may delay the recognition of myositis-associated ILD as an underlying cause of ARDS and consequently postpone appropriate treatment. In this context, our findings provide clinically relevant insights.

The patients in our study shared several clinical characteristics. In addition to fever, skin lesions were the most common extrapulmonary manifestation, occurring exclusively in patients with anti-MDA-5 or anti-Jo-1 antibodies. In contrast, patients with other MSAs infrequently exhibited these manifestations, consistent with previous reports of a lower incidence of skin lesions in non-anti-Jo-1 ASS subgroups [18,19]. Nevertheless, because hand skin lesions are readily observable, a thorough physical examination is warranted in patients with ARDS who lack obvious risk factors. All patients demonstrated elevated CRP levels, and KL-6 levels were increased in nearly all cases in which they were measured. Procalcitonin concentrations remained below 0.5 ng/mL—a level generally lower than that typically observed in bacterial pneumonia—with the exception of two patients. One had a culture-confirmed *K. pneumoniae* infection, while the other had respiratory bacterial PCR detecting *S. pneumoniae* and *H. influenzae*. Such biomarker profiles may provide supportive clues in the evaluation of ILD in this clinical context [18,20]. Seven patients had a negative ANA test; however, it is well established that patients with anti-MDA5 or ARS antibodies may exhibit weak or even negative ANA staining [21]. Accordingly, a negative ANA result does not exclude the diagnosis of myositis. Radiologic assessment revealed diffuse lesion with lower lobe predominance, most commonly manifesting as DAD or OP patterns. While OP and NSIPs are more commonly described in other cohorts of myositis-associated ILD [8,22], the predominance of DAD patterns in our study likely reflects the severity of lung injury in patients requiring ICU admission. Taken together, these clinical, laboratory, and radiologic characteristics suggest several potential clues that may assist clinicians in recognizing myositis-associated ILD in the setting of ARDS. The presence of lower lobe predominant ground-glass opacities or consolidation, in conjunction with a laboratory profile characterized by low procalcitonin, elevated CRP, and high KL-6, as well as hallmark skin manifestations involving the hands, may raise suspicion for myositis-associated interstitial lung disease. In such cases, testing for MSAs would be clinically informative.

Compared to prior research, some notable variations were observed in the clinical characteristics of myositis-associated ILD patients in this study. Myositis-associated ILD has historically been considered an uncommon condition, and only a limited number of investigations have examined patients with acute respiratory failure, including one study that analyzed 47 cases admitted to 35 ICUs in France over a 13-year period [18]. In contrast, our study identified 10 patients over a 3-year period at a single center. Although the absolute number of patients was limited, the accumulation of 10 cases over a 3-year period at a single center suggests that myositis-associated ILD presenting as ARDS may be encountered in clinical practice and warrants heightened awareness in critical care settings.

Another notable finding of our study was the high mortality rate, with 60% of patients dying, reflecting the severity of acute presentations observed in this cohort. Consistent with this observation, a multicenter study from France evaluating patients with myositis-associated ILD who presented with acute respiratory failure reported a hospital mortality rate of 51% [18], whereas studies including patients with a more chronic disease course have shown 1-year survival rates of approximately 90% [8,23]. Those studies also demonstrated poor outcomes among patients with an acute onset [23], and myositis-associated ILD showed a higher proportion of acute-onset presentations than other CTD-ILDs [8]. Taken together, our data and prior reports indicate that myositis-associated ILD can present acutely and is associated with high mortality, underscoring the importance of considering it as a potential underlying cause of ARDS.

These poor outcomes may be related to several factors. In our study, all patients who required escalation from HFNC to mechanical ventilation ultimately died. Progression from HFNC to mechanical ventilation may reflect greater baseline severity as well as a deteriorating clinical course. A similar pattern of therapeutic escalation was observed in the pharmacological regimens employed. Current guidelines recommend steroid pulse therapy combined with one or two immunosuppressive agents in cases of rapidly progressive ILD associated with CTD [24]. In contrast, while high-dose corticosteroids were administered to all patients, only six individuals received pulse therapy. It is noteworthy that among patients who received steroid pulse therapy, all patients in whom pulse therapy was added after initial high-dose corticosteroid treatment ultimately died. Additional immunosuppressive therapies were introduced sequentially after corticosteroid treatment. Taken together, these observations suggest that delayed recognition of myositis-associated ILD in patients presenting with ARDS may narrow the window for timely implementation of immunomodulatory therapy. However, this interpretation should be approached with caution, as late initiation of intensive immunomodulatory therapy may also reflect progressive clinical deterioration and the use of these treatments as rescue interventions rather than a direct effect of treatment timing.

This study has several limitations. First, the number of patients was limited, restricting the analysis to descriptive observations without formal statistical comparisons. This study contributes to the characterization of myositis-associated ILD presenting as ARDS in a critical care setting. Second, as this is a single-center retrospective study, it is subject to inherent biases, and the results may not be generally applicable. Third, the decision to perform MSA testing was based on the attending physicians’ clinical judgment. In clinical practice, testing was generally considered when ILD was suspected on imaging in patients with acute respiratory failure. Therefore, some cases without radiologic features suggestive of ILD may have remained unrecognized, potentially introducing selection bias and limiting the generalizability of our findings. More systematic approaches to antibody testing may facilitate the identification of myositis-associated ILD in patients presenting with ARDS. Fourth, three patients had positive *Pneumocystis jirovecii* PCR results, raising the possibility that *Pneumocystis* infection may have contributed to the development of ARDS. However, β-D-glucan levels were negative in the two patients in whom the test was performed, making active *Pneumocystis* pneumonia less likely. Nevertheless, because complete exclusion is difficult in critically ill patients, all three patients received antimicrobial therapy. Among these patients, two died and one ultimately underwent lung transplantation. However, because all three patients were also positive for anti-MDA5 antibodies, which are associated with poor prognosis, the clinical outcomes should be interpreted cautiously.

## 5. Conclusions

In ARDS patients without a clear etiology, myositis-associated ILD may be considered as a potential differential diagnosis. Careful physical examinations, particularly with attention to cutaneous findings, may assist in raising clinical suspicion. A negative ANA test does not exclude CTD, and assessment for MSAs may aid in the diagnostic evaluation of ARDS of unclear etiology. When myositis-associated ILD is suspected, reliance on strict classification criteria alone may delay clinical decision-making, and early therapeutic consideration may be reasonable in the appropriate clinical context.

## Figures and Tables

**Table 1:** Demographic and clinical features.

Patient	MSA	Age/Sex	Smoking	Comorbidity	Respiratory Symptom	Extrapulmonary Symptom
1	PL-7	72/M	Current smoker	None	Dyspnea, cough, sputum	None
2	MDA-5	58/F	Never smoker	None	Dyspnea, cough, sputum	Fever, skin lesions
3	Jo-1	67/M	Never smoker	MI	Dyspnea, cough, sputum	Fever, skin lesions, myalgia muscle weakness, arthralgia
4	MDA-5 Mi-2 alpha	57/F	Never smoker	None	Dyspnea, cough, sputum chest pain	Fever
5	EJ	78/M	Never smoker	MI, cirrhosis	N/A	Fever
6	MDA-5	59/M	Never smoker	Atrial fibrillation	Dyspnea, cough	Myalgia, muscle weakness
7	MDA-5	60/M	Never smoker	Diabetes	Dyspnea, cough	Skin lesions
8	PL-7	74/M	Ex-smoker	None	Dyspnea, cough	Fever
9	NXP-2	62/M	Ex-smoker	None	Dyspnea, cough, sputum hemoptysis	None
10	MDA-5	61/M	Never smoker	MI, diabetes	Dyspnea	Fever, skin lesions, myalgia

MSA, myositis-specific antibody; MDA-5, melanoma differentiation-associated gene 5; MI; myocardial infarction; N/A, not available.

**Table 2:** Laboratory findings.

Patient	WBC (/μL)	CRP (mg/L)	PCT (ng/mL)	KL-6 (U/mL)	CPK (IU/L)	LDH (IU/L)	Autoantibody *	BAL Differential Cell Count (%)
1	13,800	21.3	1.17	1501	1874	941	ANA (-); other (N/A)	MΦ (2) Lym (0) Neu (98) Eos (0)
2	6700	0.8	0.33	2528	141	1607	ANA (-); other (SSA)	MΦ (36) L (41) Neu (23) Eos (0)
3	18,500	12.9	0.14	490	1619	1459	ANA (1:40, speckled); other (SSA)	MΦ (1) Lym (1) Neu (96) Eos (2)
4	14,300	46.9	4	624	198	1485	ANA (-); other (-)	N/A
5	9300	18	0.06	1259	163	866	ANA (-); other (N/A)	MΦ (8) Lym (2) Neu (88) Eos (2)
6	15,900	0.7	0.08	N/A	292	983	ANA (-); other (N/A)	MΦ (32) Lym (20) Neu (48) Eos (0)
7	21,900	7.3	0.43	N/A	83	1784	ANA (-); other (SSA)	MΦ (1) Lym (1) Neu (98) Eos (0)
8	13,100	17.3	0.08	N/A	128	917	ANA (1:80, homogeneous nuclear); other (-)	N/A
9	13,000	9.8	0.06	558	N/A	318	ANA (1:160, speckled); other (-)	MΦ (50) Lym (5) Neu (45) Eos (0)
10	4200	9.4	0.19	1048	255	985	ANA (-); other (-)	N/A

* Autoantibody: “other (N/A)” indicates that additional specific autoantibody testing (e.g., SSA/SSB) was not performed. “other (-)” indicates that testing was performed and negative. Positive antibodies are listed by name (e.g., SSA(+)). WBC, white blood cell; CRP, C-reactive protein; PCT, procalcitonin; KL-6, Krebs von den Lungen-6; CPK, creatine phosphokinase; LDH, lactate dehydrogenase; ANA, antinuclear antibody; MΦ, macrophage; Lym, lymphocyte; Neu, neutrophil; Eos, eosinophil; N/A, not available.

**Table 3:** Microbiological evaluation.

Patient	Blood Culture	Respiratory Culture	Respiratory Bacterial/Viral PCR	Urinary Antigen Test	*Pneumocystis* PCR	β-D-Glucan (pg/mL)
1	No growth	*K. pneumoniae*	Negative	Negative	Negative	N/A
2	No growth	No growth	*H. influenzae*	Negative	Positive	38.4
3	No growth	No growth	Negative	Negative	Negative	<10
4	No growth	No growth	*S. pneumoniae* *H. influenzae*	Negative	Negative	N/A
5	No growth	No growth	Negative	Negative	Negative	N/A
6	No growth	No growth	Negative	Negative	Positive	N/A
7	No growth	No growth	Negative	Negative	Negative	N/A
8	No growth	No growth	Negative	Negative ^#^	Negative	N/A
9	No growth	No growth	Negative **	Negative	Negative	N/A
10 *	No growth	No growth	Negative	Negative	Positive	<10

* Respiratory specimen was obtained from sputum without bronchoscopy. ** Respiratory viral PCR assay was not performed. ^#^ Legionella urinary antigen test was not performed. PCR, polymerase chain reaction; N/A, not available.

**Table 4:** Radiologic findings.

Patient	Reported Findings	Predominantly Involved Region	ILD Pattern
1	Ground-glass opacity, consolidation	Both lungs	DAD
2	Ground-glass opacity, reticulation	Both lungs in peripheral dominancy	DAD
3	Ground-glass opacity, consolidation, reticulation, traction bronchiectasis	Both lungs in lower lobe dominancy	DAD
4	Ground-glass opacity, consolidation	Both lungs in lower lobe dominancy	DAD
5	Ground-glass opacity, consolidation, reticulation, traction bronchiectasis, honeycomb	Both lungs in lower lobe dominancy	DAD
6	Ground-glass opacity, consolidation	Both lungs in peripheral dominancy	OP/NSIP
7	Ground-glass opacity, consolidation, pneumomediastinum	Both lungs in lower lobe dominancy	DAD
8	Ground-glass opacity, consolidation, reticulation	Both lungs in peripheral dominancy	OP/NSIP
9	Ground-glass opacity, consolidation, reticulation	Both lungs in lower lobe dominancy	OP
10	Ground-glass opacity	Both lungs in lower lobe dominancy	OP/NSIP

ILD, interstitial lung disease; DAD, diffuse alveolar damage; OP, organizing pneumonia; NSIP, nonspecific interstitial pneumonia.

**Table 5:** Management and outcomes.

Patient	Initial Respiratory Support	P/F Ratio (mmHg)	SOFA Score	Escalation of Respiratory Support	Immunosuppressive Agents	Mortality
1	IMV	116	7	None	mPD 250 mg for 4 days, then 1 mg/kg	Alive
2	IMV	128	7	ECMO, LT	mPD 500 mg for 3 days, then 1 mg/kg	Alive
3	HFNC	123	2	IMV	mPD 1 mg/kg; CYC 500 mg/m^2^; MMF 500 mg/day; IVIG 0.4/kg	Dead
4	HFNC	173	2	None	mPD 1 mg/kg	Alive
5	HFNC	102	4	IMV	mPD 1 mg/kg	Dead
6	HFNC	74	3	IMV	mPD 1 mg/kg with 1 g for 3 days	Dead
7	IMV	50	8	ECMO	mPD 2 mg/kg with 500 mg for 3 days	Dead
8	IMV	137	6	None	mPD 1 m/kg with 1 g for 3 days; CYC 500 mg/m^2^	Dead
9	HFNC	188	2	None	mPD 1 mg/kg; MMF 500 mg/day; TAC 4 mg/day; IVIG 0.4/kg	Alive
10	HFNC	150	2	IMV, ECMO	mPD 1 mg/kg with 500 mg for 3 days; TAC 2 mg/day	Dead

P/F ratio, ratio of the partial pressure of arterial oxygen to the fraction of inspired oxygen; SOFA score, Sequential Organ Failure Assessment; IMV, invasive mechanical ventilation; HFNC, high-flow nasal cannula; ECMO, extracorporeal membrane oxygenation; LT, lung transplantation; mPD, methylprednisolone; CYC, cyclophosphamide; MMF, mycophenolate mofetil; IVIG, intravenous immunoglobulin; TAC, tacrolimus.

## Data Availability

The original contributions presented in this study are included in the article. Further inquiries can be directed to the corresponding author.

## Abbreviations

The following abbreviations are used in this manuscript:

ARDS	Acute respiratory distress syndrome
CTD	Connective tissue disease
ILD	Interstitial lung disease
IIM	Idiopathic inflammatory myopathy
MSA	Myositis-specific antibody
ICU	Intensive care unit
HFNC	High-flow nasal cannula
ARS	Anti-aminoacyl-tRNA synthetase
MDA-5	Melanoma differentiation-associated gene 5
CT	Computed tomography
ASS	Anti-synthetase syndrome
EULAR/ACR	European League Against Rheumatism and American College of Rheumatology
CRP	C-reactive protein
ANA	Anti-nuclear antibody
BAL	Bronchoalveolar lavage
PCR	Polymerase chain reaction
DAD	Diffuse alveolar damage
OP	Organizing pneumonia
NSIP	Non-specific interstitial pneumonia
P/F ratio	Ratio of the partial pressure of arterial oxygen to the fraction of inspired oxygen
ECMO	Extracorporeal membrane oxygenation
